# Fatty Acid Oxidation Compensates for Lipopolysaccharide-Induced Warburg Effect in Glucose-Deprived Monocytes

**DOI:** 10.3389/fimmu.2017.00609

**Published:** 2017-05-29

**Authors:** Nora Raulien, Kathleen Friedrich, Sarah Strobel, Stefan Rubner, Sven Baumann, Martin von Bergen, Antje Körner, Martin Krueger, Manuela Rossol, Ulf Wagner

**Affiliations:** ^1^Division of Rheumatology, Department of Internal Medicine, University of Leipzig, Leipzig, Germany; ^2^Department of Molecular Systems Biology, Helmholtz Centre for Environmental Research – UFZ, Leipzig, Germany; ^3^Faculty of Biosciences, Pharmacy and Psychology, Institute of Pharmacy, University of Leipzig, Leipzig, Germany; ^4^Faculty of Biosciences, Pharmacy and Psychology, Institute of Biochemistry, University of Leipzig, Leipzig, Germany; ^5^Hospital for Children and Adolescents, Department of Women and Child Health, University Hospitals, University of Leipzig, Leipzig, Germany; ^6^Institute of Anatomy, University of Leipzig, Leipzig, Germany

**Keywords:** monocytes, Warburg effect, fatty acid oxidation, glucose deprivation, inflammation

## Abstract

Monocytes enter sites of microbial or sterile inflammation as the first line of defense of the immune system and initiate pro-inflammatory effector mechanisms. We show that activation with bacterial lipopolysaccharide (LPS) induces them to undergo a metabolic shift toward aerobic glycolysis, similar to the Warburg effect observed in cancer cells. At sites of inflammation, however, glucose concentrations are often drastically decreased, which prompted us to study monocyte function under conditions of glucose deprivation and abrogated Warburg effect. Experiments using the Seahorse Extracellular Flux Analyzer revealed that limited glucose supply shifts monocyte metabolism toward oxidative phosphorylation, fueled largely by fatty acid oxidation at the expense of lipid droplets. While this metabolic state appears to provide sufficient energy to sustain functional properties like cytokine secretion, migration, and phagocytosis, it cannot prevent a rise in the AMP/ATP ratio and a decreased respiratory burst. The molecular trigger mediating the metabolic shift and the functional consequences is activation of AMP-activated protein kinase (AMPK). Taken together, our results indicate that monocytes are sufficiently metabolically flexible to perform pro-inflammatory functions at sites of inflammation despite glucose deprivation and inhibition of the LPS-induced Warburg effect. AMPK seems to play a pivotal role in orchestrating these processes during glucose deprivation in monocytes.

## Introduction

Circulating monocytes in the peripheral blood are part of the first-line defense of the immune system and are among the first cells entering sites of inflammation. There, they react to a multitude of danger signals with a uniform set of effector mechanism including activation of the multiprotein complex of the inflammasome (1–3).

Activation of monocytes by lipopolysaccharide (LPS) leads to cell enlargement and an increased demand for biosynthesis of proteins, lipids, and DNA. To meet those requirements, stimulation of monocytes/macrophages by LPS is accompanied by a shift toward increased glycolytic flux, an effect similar to the Warburg effect observed in cancer cells (4, 5). In addition, upregulation of the pentose–phosphate pathway occurs, secretion of lactate increases, and intermediates in the tricarboxylic acid (TCA) cycle are in higher demand due to the necessary synthesis of amino acids and lipids (6).

Immunometabolism has recently received considerable attention. Metabolic studies in macrophages have revealed differential pathways regulated during differentiation into M1 and M2 phenotypes (7–10). Similar studies have been undertaken for T helper cell subsets (6, 11, 12), dendritic cells (13), and to a limited extent also in monocytes (5). In well-perfused inflamed tissues like the synovial membrane in rheumatoid arthritis, where nutrients are in ample supply, the massively upregulated glycolytic flux of activated monocytes and macrophages is sustained by high glucose consumption. This has been visualized best by FDG-PET, which allows the identification of inflammatory sites in cases of microbial infection or sterile autoimmune inflammation, based on the massive uptake of the glucose-analogon 2-fluoro-2-deoxy-d-glucose (in the form of the radioactive tracer ^18^F-2-FDG) (14).

Due to the upregulated glycolytic flux, a tremendous glucose supply is required at sites of active inflammation. On the other hand, many infectious and sterile inflammations have been associated with severely reduced glucose concentrations, including bacterial, mycobacterial, and fungal central nervous system (CNS) infections (15) or pleural effusions (16, 17). In addition, non-infectious inflammatory conditions can also be associated with very low glucose concentrations, including sarcoidosis (18), rheumatoid meningitis (19), and rheumatoid pleurisy (20–22), as well as CNS involvement and pleural effusion associated with systemic lupus erythematosus (15, 23). In general, any factors compromising blood flow and glucose supply—like conditions of impaired microcirculation due to septic shock, arteriosclerotic vessel damage, or vasculitis—are likely to tilt the balance of glucose supply and lead to glucose deprivation. Consequently, at such sites of severe inflammation with deficient perfusion, glucose is not freely available, and a high level of glycolysis is therefore impossible. On the other hand, glucose starvation is invariably followed by activation of AMP-activated protein kinase (AMPK), which in turn initiates multiple catabolic pathways including glucose uptake, fatty acid uptake and oxidation, mitochondrial biogenesis, and autophagy (24).

In this study, we investigated cellular and immunologic parameters in the LPS-induced immune response of monocytes under conditions of glucose deprivation, in order to identify crucial metabolic pathways and checkpoints that must be sustained. We found that in the absence of glucose and without glycolysis, monocytes rely mainly on mitochondrial metabolism of lipids to maintain levels of ATP, NADH, and NADPH and are still able to fulfill their immunological pro-inflammatory function. Functional limitation in the absence of glucose is a decreased—though not abrogated—respiratory burst, which is likely a protective measure by the starved monocyte.

## Materials and Methods

### Antibodies and Reagents

Where not stated otherwise, monocytes were cultured in glucose-free RPMI 1640 (#11879, Gibco, Thermo Fisher) supplemented with 2 mM GlutaMAX (Gibco, Thermo Fisher), 10% heat-inactivated FCS (Gibco, Thermo Fisher), and 5 mM glucose (Sigma-Aldrich) or without glucose. PBS was purchased from Biochrom (Merck Millipore). The following antibodies were used for Western Blot: anti-phosphoAMPKα Thr172 (40H9) rabbit monoclonal antibody (#2535, Cell Signaling), anti-AMPKα (F6) mouse monoclonal antibody (#2793, Cell Signaling), anti-β-actin (D6A8) rabbit monoclonal antibody (#8457, Cell Signaling), anti-rabbit-HRP goat antibody (sc-2056, Santa Cruz Biotech.), anti-mouse-HRP goat antibody (sc-2055 Santa Cruz Biotech.), and anti-LC3 (APG 8) monoclonal mouse antibody (#AM1800a, Abgent, USA). Fluorescence microscopy was done with the antibodies anti-LC3 antibody polyclonal rabbit (#PM036, MBL, USA) and anti-rabbit-Alexa Fluor 594 goat antibody (#111-585-003, Jackson Immunoresearch, USA).

### Monocyte Isolation, Cell Culture, and Cytokine Measurement

This study was carried out in accordance with the recommendations of “Ethik-Kommission an der Medizinischen Fakultät der Universität Leipzig” with written informed consent from all blood donors. The protocol was approved by the aforementioned committee. Monocytes were isolated as described previously (25). For cytokine analysis, 3 × 10^5^ monocytes per 200 µl RPMI 1640 with 5 mM glucose or without glucose were cultured at 37°C and 5% CO_2_ in 96-well plates. Four and sixteen hours after stimulation with 100 ng/ml LPS, supernatants were removed and analyzed by ELISA for cytokines IL-1β, TNF, IL-6, IL-8 (all BD OptEIA, Becton Dickinson), IL-1α and MCP-1 (both DuoSet ELISA, R&D Systems) following the instructions of the assay.

### Metabolism Analysis

Glycolytic and mitochondrial activity of monocytes was measured using a Seahorse extracellular flux 96 analyzer (Seahorse Bioscience). Monocytes were seeded at a density of 4 × 10^5^ cells/100 µl RPMI1640 (#61870, Gibco, Thermo Fisher) without FCS and adhered for 30 min at cell culture conditions. After two washing steps, 180 µl RPMI 1640 (R1383, Sigma-Aldrich) containing 5% FCS, 2 mM glutamine, and 5 mM glucose or without glucose was added. The injection ports were filled with RPMI 1640 without FCS plus the indicated stimuli or inhibitor (LPS-EB ultrapure from *E. coli* 0111:B4 strain 100 ng/ml, Invivogen; apocynin 1 mM, Sigma-Aldrich; 2-deoxyglucose (2-DG) 5 mM, Sigma-Aldrich; etomoxir 250 µM, Sigma-Aldrich; BPTES 30 µM, Sigma-Aldrich; dorsomorphin/compound C 10 µM, Abcam). Extracellular acidification rate (ECAR) and oxygen consumption rate (OCR) were determined at 37°C. The parameters of oxidative phosphorylation were analyzed following the injection of the inhibitors oligomycin (1 µM; Cayman Chemicals), FCCP (2 µM; Cayman Chemicals), Rotenone (1 µM; Sigma-Aldrich), and antimycin A (1 µM; Sigma-Aldrich).

### Measurement of intracellular Polar Metabolites Using IC-MS/MS

Monocytes (1 × 10^6^/700 µl) were stimulated with 100 ng/ml LPS in the presence of 5 mM glucose or in glucose-deficient media on agarose-coated wells. Cells were harvested and centrifuged (350 × *g*, 5 min) 1, 3, and 6 h after stimulation and washed one time with PBS. The pellet was dissolved in 500 µl 45% (v/v) methanol/5% (v/v) chloroform. After the addition of 500 µl water the suspension was mixed 30 min at 4°C. The solution was centrifuged at 500 × *g* for 10 min, the upper phase was transferred and vacuum dried. For ion chromatography-tandem mass spectrometry (IC-MS/MS)-based analysis of the metabolites, dried extracts were redissolved in 300 µl water. Samples were afterward up to another 10-fold diluted, and a total volume of 25 µl was analyzed on an ICS-5000 (Thermo Fisher Scientific, Dreieich, Germany) coupled to an API 5500 QTrap (AB Sciex) as described elsewhere (26). Separation was achieved on an IonPac AS11-HC column (2 mm × 250 mm, Thermo Fisher Scientific) with an increasing potassium hydroxide gradient. MS analysis was performed in multiple reaction monitoring (MRM) mode using negative electrospray ionization and included organic acids and carbohydrates involved in central metabolite pathways. Metabolites were considered to be detectable above a signal-to-noise ratio (S/N) of three within a retention time window of 0.5 min.

### Measurement of Lipid Content Using Biocrates IDQ P150

Cellular extracts were analyzed using the “AbsoluteIDQ p150 Kit” (Biocrates, Innsbruck, Austria). Briefly, samples were prepared as follows: (a) pipetting of 10 µl cell extract onto the filter inserts of the 96-well plate of the kit (containing internal standards labeled with stable isotopes), (b) drying of samples under a nitrogen stream, (c) extraction of metabolites and internal standards with 5 mM ammonium acetate in methanol, (d) centrifugation through the membrane filter provided, and (e) dilution with solvent for mass spectrometry. The final extracts were then randomized and analyzed using a 4000 QTrap mass spectrometer (Sciex) equipped with a TurboIonSpray source and coupled to an Agilent 1100 Series HPLC. Standard flow injection comprised two 20 µl injections (one for positive and one for negative electrospray ionization mode), and MRM was used for quantification as described earlier (27). Data were subsequently analyzed using the MetIQ software package (Biocrates, Innsbruck, Austria).

### Intracellular AMP and ATP Determination

The intracellular AMP and ATP concentration was measured in cells (3 × 10^5^/200 µl in 96-well plate) cultured for 1, 3, and 16 h with LPS stimulation (100 ng/ml) and glucose or in glucose-deprived medium. Culture plates were centrifuged (400 × *g*, 5 min), supernatant was discarded, and cells were lysed in 700 µl ATP-Lysis buffer [100 mM potassium phosphate buffer (pH 7.8), 1 mM DTT, 2 mM EDTA, 1% Triton X-100 (v/v)]. ATP concentrations in the lysate were determined with the ATP Determination Kit (Thermo Fisher), and AMP was measured with the AMP-Glo Assay (Promega) following the instructions. Resulting concentrations were normalized to protein concentrations of the lysate determined with DC-Protein Assay from BioRad.

### Intracellular NADH and NADPH Determination

Intracellular levels of NADH and NADPH were determined with the PicoProbe NADH Quantitation Fluorometric Kit and the PicoProbe NADPH Quantitation Fluorometric Kit (BioVision) following the manual instructions. After removal of the cell supernatant, 3 × 10^5^ monocytes were lysed in 150 µl extraction buffer. Concentrations of NADH and NADPH were calculated using standard curves and normalized to total protein concentration of the lysate.

### Lactate-Dehydrogenase Cytotoxicity Assay

The cellular vitality was determined with the Pierce LDH Cytotoxicity Assay Kit (Thermo Scientific). Cells were seeded in duplicate (3 × 10^5^ cells/200 µl) in a 96-well plate. Lactate dehydrogenase (LDH) was determined in the supernatant and in lysed cells [addition of 20 µl 9% Triton X-100 (Serva, Heidelberg, Germany) solution] following the assay instructions. Cytotoxicity was calculated with the following equation:

$$\%\text{ cytotoxicity}=\frac{\text{LDH activity supernatant}*100\%}{\text{LDH activity supernatant of lysed cells}}.$$

### Chemotactic Migration Assay

The chemotactic migration of monocytes was analyzed with the InnoCyte Monocyte Cell Migration Assay (Merck Millipore, Darmstadt, Germany). A total of 3 × 10^5^ cells/100 µl were stimulated with 100 ng/ml LPS in the absence or presence of glucose and applied into the upper chamber of the assay plate. The lower chamber was filled with complete medium (±glucose) containing 100 ng/ml MCP-1 (Miltenyi, Bergisch Gladbach, Germany). After 4 h, the plates were separated, and the cells in the lower compartment were stained with calcein AM. The fluorescence was measured using a plate reader at excitation 485 nm and emission 520 nm.

### Phagocytosis Assay

Phagocytic activity of monocytes was determined with pHrodo Green *E. coli* BioParticle conjugates (P35366, Molecular Probes). Monocytes were seeded at a density of 6 × 10^5^ cells/200 µl in an agarose-coated 48-well plate and stimulated with 100 ng/ml LPS. After 2 h of incubation 50 µg bacterial particles were added for 10 min. The fluorescence of monocytes was determined with a FACSCalibur (Becton Dickinson).

### Measurement of the Oxidative Burst

Monocytes were resuspended in phenol red free SILAC RPMI 1640 (Gibco, Thermo Fisher) supplemented with 2 mM GlutaMAX (Gibco, Thermo Fisher), 5 mM HEPES (Sigma-Aldrich), 0.133 mM l-arginine (Sigma-Aldrich), 0.27 mM l-lysine (Sigma-Aldrich), and 2.5% FCS in a density of 3 × 10^5^/200 µl and seeded in a white 96-well plate (Nunc, Thermo Fisher). After 2 h of incubation at 37°C and 5% CO_2_, luminol reagent (140 µM; Cayman Chemicals) was added, and cells were stimulated with 100 ng/ml LPS. Luminescence from oxidized luminol was measured with a platereader (FluoStar Optima, BMG Labtech) over 180 min. Activation of the AMPK with 1 mM AICAR (Sigma-Aldrich) was done prior to LPS stimulation.

### LC3 Fluorescence Microscopy in THP1 Cells

THP1 cells (1.8 × 10^5^) were differentiated for 2 days with 100 ng/ml PMA (Sigma-Aldrich) on glass slides in a 24well plate in RPMI 1640 (#61870 Gibco, Thermo Fisher; 10% FCS, 1% penicillin/streptomycin). After washing with PBS, the cells were stimulated with 100 ng/ml LPS in RPMI 1640 (10% FCS) with 5 mM glucose or without glucose for 3 h. Cells were immediately fixed with 4% paraformaldehyde (10 min, 4°C) and permeabilized with 0.1% Triton X-100 for 10 min. The nucleus was stained with 3 µg/ml Hoechst (Invitrogen, Thermo Fisher), and after blocking (10% BSA; 1 h), LC3 antibody was incubated over night at 4°C. Unbound antibody was removed in washing steps with PBS, and the secondary antibody was incubated for 1 h. Slides were embedded and analyzed with an Axiostar microscope (Zeiss).

### Western Blot

For determination of LC3-I and LC3-II by Western Blot, 3 × 10^6^ cells/ml were stimulated for 3 h with 100 ng/ml LPS in RPMI 1640 with or without glucose in the presence of 30 mM NH_4_Cl. Cells were washed with PBS and immediately lysed in 100 µl Laemmli-buffer (62.8 mM Tris–HCl, 11.8% glycerol, 2% SDS, 102 mM DTT, 0.01% bromophenol blue). The lysate was sonicated (four pulses for 2 s each, 30% amplitude), boiled for 5 min, and loaded on a 15% Tris–Tricine–SDS-PAGE. Electroblotting on polyvinylidene difluoride membrane, antibody incubation, visualization, and the immunoblot of phospho AMPKα (Thr172) was done as described previously (25). Densitometric analysis was performed using ImageJ software.

### Scanning Transmission Electron Microscopy

Monocytes (1.5 × 10^6^) were stimulated with 100 ng/ml LPS in RPMI 1640 with 5 mM glucose or without glucose on collagen-coated slides (Thermanox, Nunc) in a 24-well plate. For electron microscopy, samples were fixed using 4% paraformaldehyde (Serva, Heidelberg, Germany) and 1% glutaraldehyde (Serva) in PBS followed by staining with 0.5% osmium tetroxide (EMS, Hatfield, PA, USA). After thorough rinsing in PBS, the sections were dehydrated in graded alcohol and further stained with 1% uranyl acetate (Merck, Darmstadt, Germany) in 70% alcohol. After final dehydration, the samples were transferred in propylene oxide (Sigma-Aldrich, Steinheim, Germany) and incubated in Durcupan (Sigma-Aldrich). After polymerization at 56°C for 48 h, the cell culture insert was removed, and the blocks of resin were trimmed and finally cut using an ultramicrotome (Leica Microsystems, Wetzlar, Germany). Ultrathin sections with an average thickness of 55 nm were transferred on formvar-coated copper grids and stained with lead citrate. Analysis was performed using a Zeiss SIGMA electron microscope (Zeiss NTS, Oberkochen, Germany) equipped with a STEM detector and ATLAS software. Monocytes were single-blinded analyzed for lipid droplets (LDs) in the cytosol. For each condition, time point and donor (*n* = 3), 15 microscopic pictures were taken, and all cells with nucleus (28–107 per condition) were classified into LD positive or negative.

### Statistical Analysis

For statistical analysis, GraphPad Prism was used. Student’s paired or unpaired *t*-test was used where appropriate and indicated.

## Results

### Glucose Deprivation of LPS-Activated Monocytes Abrogates Aerobic Glycolysis and Leads to a Compensatory Increase of Oxidative Phosphorylation

**Figure 1:**
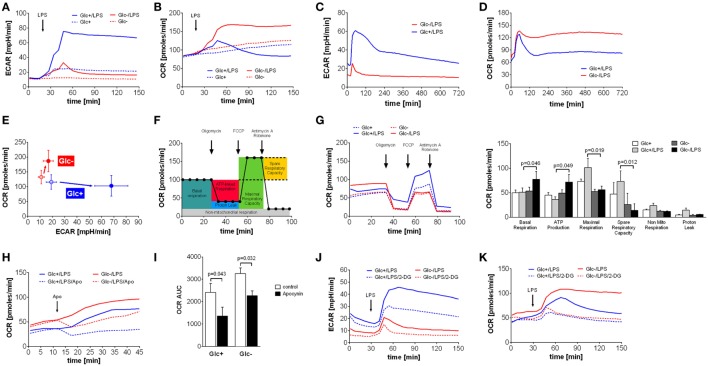
**Abrogation of lipopolysaccharide (LPS)-induced Warburg effect by glucose deprivation in monocytes leads to compensatory increase of oxidative phosphorylation**. **(A,B)** Shown are mean (*n* = 6) **(A)** extracellular acidification rate (ECAR) and **(B)** oxygen consumption rate (OCR) of monocytes in glucose medium or in glucose-deprived medium, measured by the Seahorse XF96 analyzer. Cells were stimulated with LPS or medium control at *t* = 20 min. **(C,D)** Long-term metabolic measurement [**(C)** ECAR and **(D)** OCR] of LPS-stimulated (*t* = 15 min) monocytes (*n* = 4). **(E)** The phenogram of ECAR and OCR rates shows mean values ± STD (*n* = 6) at *t* = 150 min generated from data sets shown in panels **(A,B)**. **(F)** Scheme of mitochondrial metabolic parameter calculation using the Mitochondrial Stress Test. **(G)** Mitochondrial Stress Test of monocytes treated for 2 h with LPS or control and with or without glucose. Changes of OCR after injection of Mitochondrial Stress Test components are shown as mean rates of *n* = 5 donors. **(H)** The contribution of NADPH oxidase to the OCR was analyzed by injection of the specific inhibitor apocynin. Shown are mean OCR rates of *n* = 3 donors. **(I)** Area under the curve of OCR rates shown in panel **(H)** was calculated and is depicted as mean ± SEM of *n* = 3 experiments. **(J,K)** The metabolic changes in LPS-treated monocytes due to glucose deprivation and inhibition of glycolysis with 2-deoxyglucose (5 mM; dashed line) are shown as ECAR **(J)** and OCR **(K)** of *n* = 4 donors. For each donor and condition, the analysis was run at least in triplicates. Statistical analysis was performed using the paired Student’s *t*-test.

Monocytes, macrophages and dendritic cells undergo a switch from oxidative phosphorylation to glycolysis in response to toll-like receptor 4 activation by LPS, the so called Warburg effect (5, 10, 28, 29). The switch to glycolysis is accompanied by increased glucose uptake and turnover (28). Therefore, we sought to determine how primary monocytes compensate for glucose deprivation during activation with LPS. Primary monocytes stimulated with LPS under standard tissue culture conditions (RPMI 1640 medium containing 5 mM glucose, 2 mM l-glutamine, and 5% FCS) exhibited a steep and prolonged increase in lactate production, as measured by ECAR using Seahorse extracellular flux analysis (Figure 1A). Monocytes incubated in glucose-deficient medium responded to LPS with a short-lived and flat increase in lactate production, presumably fueled by intracellular glucose and traces of glucose (<0.2 mM) introduced with the FCS (Figures 1A,C). In contrast to monocytes in glucose medium, however, LPS-stimulated monocytes in glucose-deprived medium exhibited an intense and prolonged increase in oxidative phosphorylation, as measured by the OCR (Figure 1B). This increased OCR is stable for up to 12 h (Figure 1D). The phenogram in Figure 1E illustrates the LPS-induced switch of monocytes to aerobic glycolysis in the presence of glucose, and the compensatory increase in oxidative phosphorylation in its absence.

Next, we sought to study the increased oxidative phosphorylation of monocytes in glucose-deprived medium in more detail using the Seahorse Mito Stress Test. The successive addition of respiration chain inhibitors and uncouplers allows the determination of basal respiration, maximal respiration, ATP production, spare respiratory capacity, non-mitochondrial respiration, and proton leak of mitochondria (Figure 1F). Analysis of monocytes stimulated with LPS in glucose-free media revealed that basal respiration and ATP production are significantly increased compared to glycolytic monocytes (Figure 1G). Maximal respiration and spare capacity are decreased in glucose-deprived monocytes, indicating maximal utilization of mitochondrial capacity (Figure 1G). Non-mitochondrial respiration and proton leak are more pronounced in LPS-activated monocytes in the presence of glucose (Figure 1G).

Non-mitochondrial respiration includes oxygen consumption during the respiratory burst in response to LPS in monocytes. As seen in Figure 1D, glycolytic monocytes also respond with an initial OCR increase to LPS, albeit with much shorter duration. This OCR peak might represent the oxygen consumption of the respiratory burst. To evaluate the contribution of the NADPH oxidase-dependent respiratory burst to the oxygen consumption leading to the observed OCR increase, apocynin, an inhibitor of the NADPH oxidase, was used. As shown in Figure 1H, apocynin partially inhibited the LPS-induced OCR increase in monocytes in both glucose-containing medium and in glucose-deprived medium. However, NADPH oxidase-dependent oxygen consumption of LPS-stimulated monocytes in glucose medium represents 46% of total oxygen consumption during this time period, but only 30% of total oxygen consumption in glucose-deprived monocytes (Figure 1I). The corresponding control experiments investigating the influence of apocynin on the oxygen consumption in unstimulated monocytes are shown in Figure S1A in Supplementary Material. The specific inhibition of the oxidative burst in monocytes with apocynin is demonstrated in Figure S1B in Supplementary Material.

2-Deoxyglucose is a widely used inhibitor of glycolysis. Therefore, we tested if the effect of 2-DG is comparable to glucose deprivation. As shown in Figure 1J, 2-DG only partially inhibited the LPS-induced switch to glycolysis of monocytes in glucose medium. More interestingly, 2-DG was not able to induce the intense OCR increase seen in monocytes in glucose-deprived medium. Instead, the addition of 2-DG to glucose-deprived monocytes revealed an inhibitory effect on the OCR, most likely due to unspecific effects of 2-DG (Figure 1K). Consequently, we used glucose deprivation in all subsequent experiments, because inhibition of glycolysis by 2-DG appears to involve other mechanisms in monocytes than glucose deprivation.

**Figure 2:**
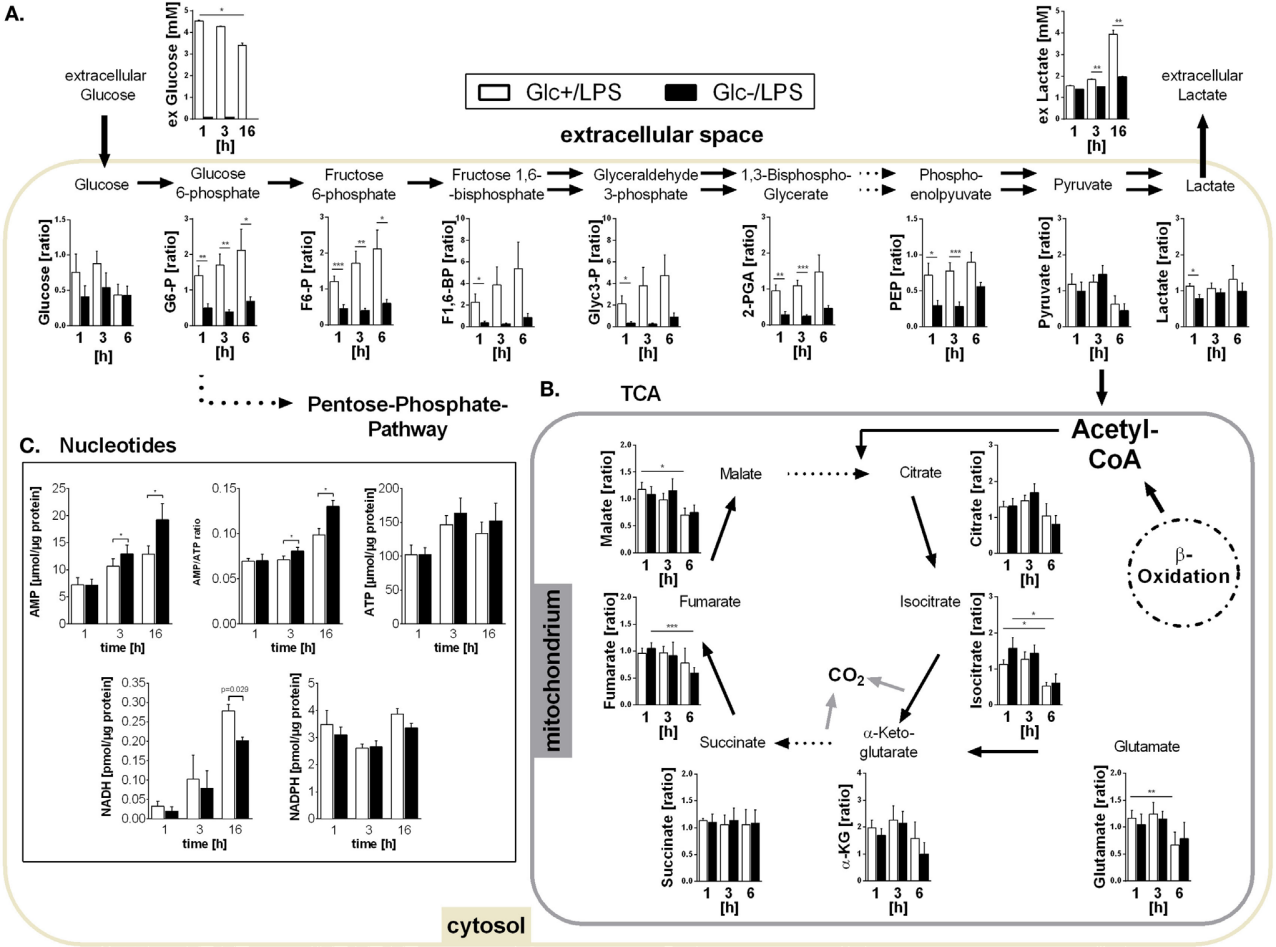
**Metabolites of glycolysis and tricarboxylic acid (TCA) and intracellular nucleotides in glucose-deprived lipopolysaccharide (LPS)-stimulated monocytes**. **(A)** Intracellular glycolytic metabolites were extracted from LPS-stimulated monocytes with methanol/chloroform and measured using IC-MS/MS. Peak areas were normalized to unstimulated freshly isolated cells. Shown are mean values of *n* = 7 (±SEM) experiments from three different donors. Extracellular glucose and lactate concentrations were determined in the supernatant of LPS-stimulated monocytes under glucose or glucose-deprived conditions at time points 1, 3, and 16 h. Shown are mean values ± SEM of *n* = 3 donors. Statistical analysis was performed using the paired Student’s *t*-test. **(B)** Intracellular TCA metabolites were generated as in panel **(A)**. **(C)** Intracellular ATP, AMP, NADH, and NADPH concentrations were determined at 1, 3, and 16 h in glycolytic or glucose-deprived monocytes stimulated with LPS. The concentrations were normalized to the whole protein content of the lysate. Bar charts show mean concentrations (±SEM) of *n* = 6 (ATP, AMP, NADPH) or *n* = 3 (NADH). Statistical analysis was performed using the paired Student’s *t*-test.

In the presence of glucose, LPS stimulation triggered an increased glucose uptake compared to resting control cells (3 h: 0.73 vs. 0.57 mM, *p* = 0.038, 16 h: 1.60 vs. 0.57 mM, *p* = 0.007) confirming the switch to increased glycolysis in response to LPS. The targeted assessment of metabolites of the central carbon metabolism of monocytes activated with LPS in the absence of glucose revealed that intracellular glucose and all intermediates of glycolysis are significantly diminished when compared to standard glucose concentrations (Figure 2A). The glycolytic end-product pyruvate, however, is present in equal amounts under both conditions (Figure 2A). It is known that the switch to aerobic glycolysis results in the conversion of pyruvate to lactate instead of oxidation in mitochondria (30). Accordingly, we observed an increased lactate secretion of LPS-activated glycolytic monocytes compared to unstimulated control cells in direct measurements of lactate in the culture supernatant (3 h: 1.83 vs. 1.57 mM, *p* = 0.057, 16 h: 3.93 vs. 1.87 mM, *p* = 0.007), which corresponded to the ECAR measurement using the Seahorse flux analyzer (see Figure 1A). Glucose-deprived, LPS-stimulated monocytes, in contrast, showed a significant reduction of both lactate secretion and intracellular lactate concentrations (Figure 2A).

As shown in Figures 1B,D, glucose-deprived monocytes responded to LPS with an increase in oxidative phosphorylation. The TCA cycle is essential for providing NADH for the electron transport chain to synthetize ATP in this process. Analysis of TCA metabolites revealed that monocytes stimulated with LPS in the presence or absence of glucose did not differ in TCA metabolite concentrations (Figure 2B). Overall, most metabolites are found in lower concentrations 6 h after LPS stimulation compared to earlier time points (Figure 2B).

The energy status of activated cells is determined by the concentrations and the ratio of the adenine nucleotides AMP and ATP. Despite increased oxidative phosphorylation, glucose-deprived monocytes had increased AMP concentrations and increased AMP/ATP ratios while ATP concentrations did not differ from LPS-stimulated glycolytic monocytes (Figure 2C). In addition, the concentration of NADPH in LPS-stimulated monocytes was not influenced by the availability of glucose. In contrast, NADH was found decreased in glucose-deprived monocytes after 16 h (Figure 2C).

### Oxidative Metabolism Is Mainly Fueled by Fatty Acids in Glucose-Deprived Monocytes

**Figure 3:**
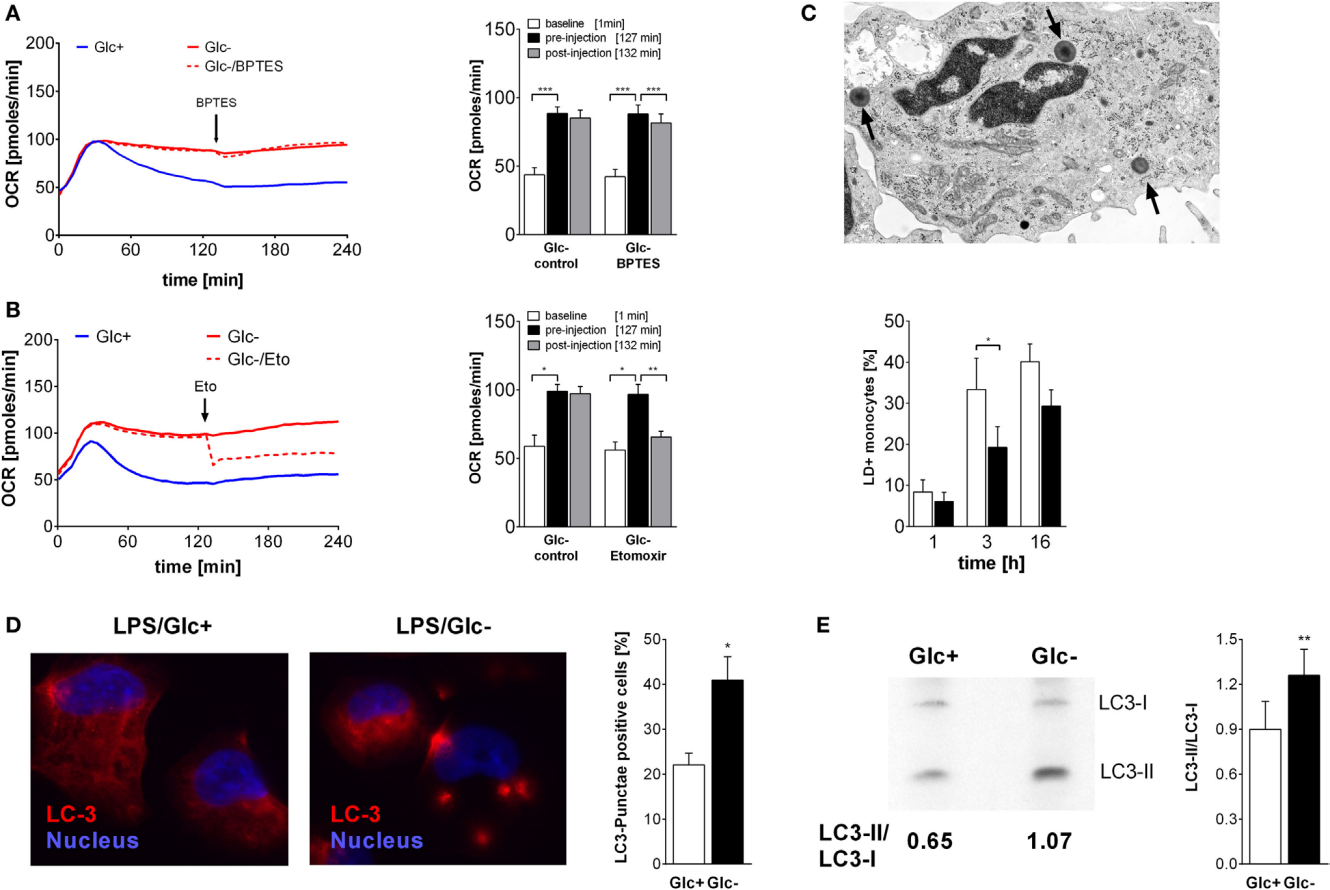
**Oxidative metabolism is fueled by glutamine and fatty acids in glucose-deprived monocytes**. **(A,B)** The oxygen consumption rate (OCR) changes of lipopolysaccharide (LPS)-stimulated monocytes following inhibition of **(A)** glutaminase with BPTES (30 µM; *n* = 6) or **(B)** fatty acid transport with etomoxir (250 µM; *n* = 4) were analyzed. The OCR is shown as mean curve over 240 min and in the bar chart as mean ± SEM at baseline (1 min), before (127 min), and after inhibitor injection (132 min). For each donor and condition, the analysis in panels **(A,B)** was run at least in duplicates. **(C)** LPS-simulated monocytes with lipid droplets (LDs; arrow) were determined with TEM at 1, 3, and 6 h. Shown is the mean ± SEM of LD-positive monocytes out of >190 cells analyzed from *n* = 3 donors. **(D)** Autophagy was analyzed in PMA-differentiated THP1 cells. Cells were stimulated with LPS with or without glucose for 3 h and stained with anti-LC3 antibody. Autophagy-positive cells (≥3 LC3 punctae/cell) were analyzed in >40 cells (*n* = 4; mean ± SEM). **(E)** Autophagy in primary LPS-stimulated monocytes with or without glucose was analyzed by Western Blot. The ratio of autophagosomal membrane-bound LC3-II to unbound LC3-I was determined after 3 h (*n* = 6; mean ± SEM). Statistical analysis was performed using the paired Student’s *t*-test.

In addition to glucose, cells use fatty acids and amino acids to fuel the TCA. Glutamine is a major substrate of macrophages (31) and is present in the culture medium (2 mM). Glutamine is converted to glutamate by the enzyme glutaminase, and subsequently deaminated to α-ketoglutarate, which fuels the TCA. The glutaminase inhibitor BPTES was used to investigate the contribution of glutamine to monocyte energy expenditure. Inhibition of glutaminase by BPTES had an only marginal influence on the increased oxygen consumption in LPS-stimulated glucose-deprived monocytes (Figure 3A).

Fatty acids are converted to acetyl-CoA and NADH in the β-oxidation. In order to test whether fatty acids are required for the oxidative phosphorylation seen in LPS-stimulated, glucose-deprived monocytes, an inhibitor of the carnitine palmitoyltransferase 1 (CPT-1) was used. CPT-1 is responsible for the transport of long-chain fatty acids into the mitochondria. Inhibition of CPT-1 using etomoxir prevented the increase of oxygen consumption induced by glucose deprivation (Figure 3B). In the presence of glucose, etomoxir also partially inhibited oxidative phosphorylation in LPS-stimulated glycolytic monocytes (data not shown).

Intracellular neutral lipids are stored in LDs, which are located in the cytoplasm of cells. We determined the presence of LDs in the cytoplasm of LPS-stimulated monocytes using electron microscopy. As shown in Figure 3C, the frequency of LD+ monocytes increases with time following LPS stimulation, and glucose deprivation decreases the frequency of LD+ monocytes. Decreased lipid synthesis following cellular starvation also results in decreased synthesis of phospholipids. Therefore, we evaluated the phosphatidylcholine content of monocytes using the Biocrates platform. We observed lower concentrations of several phosphatidylcholine species in glucose-deprived monocytes compared to glycolytic monocytes (Table S1 in Supplementary Material).

Glucose starvation is a known activator of autophagy, which is another pathway that provides nutrients by degradation of cellular components (32, 33). As expected, glucose deprivation also led to the formation of LC3 punctae in the PMA-differentiated monocytic cell line THP1 (Figure 3D) and to an increase in the LC3-II/LC3-I ratio in LPS-activated monocytes in the absence of glucose, indicating an increased autophagy compared to glycolytic monocytes (Figure 3E).

In summary, primary monocytes switch to glycolysis upon LPS stimulation in the presence of glucose. In glucose-deprived monocytes, metabolism switches to increased oxidative phosphorylation, which is fueled by fatty acids. However, the increased oxidative phosphorylation cannot completely compensate glucose deprivation, because AMP increases and NADH decreases.

### Impact of Glucose Deprivation on Monocyte Effector Functions

**Figure 4:**
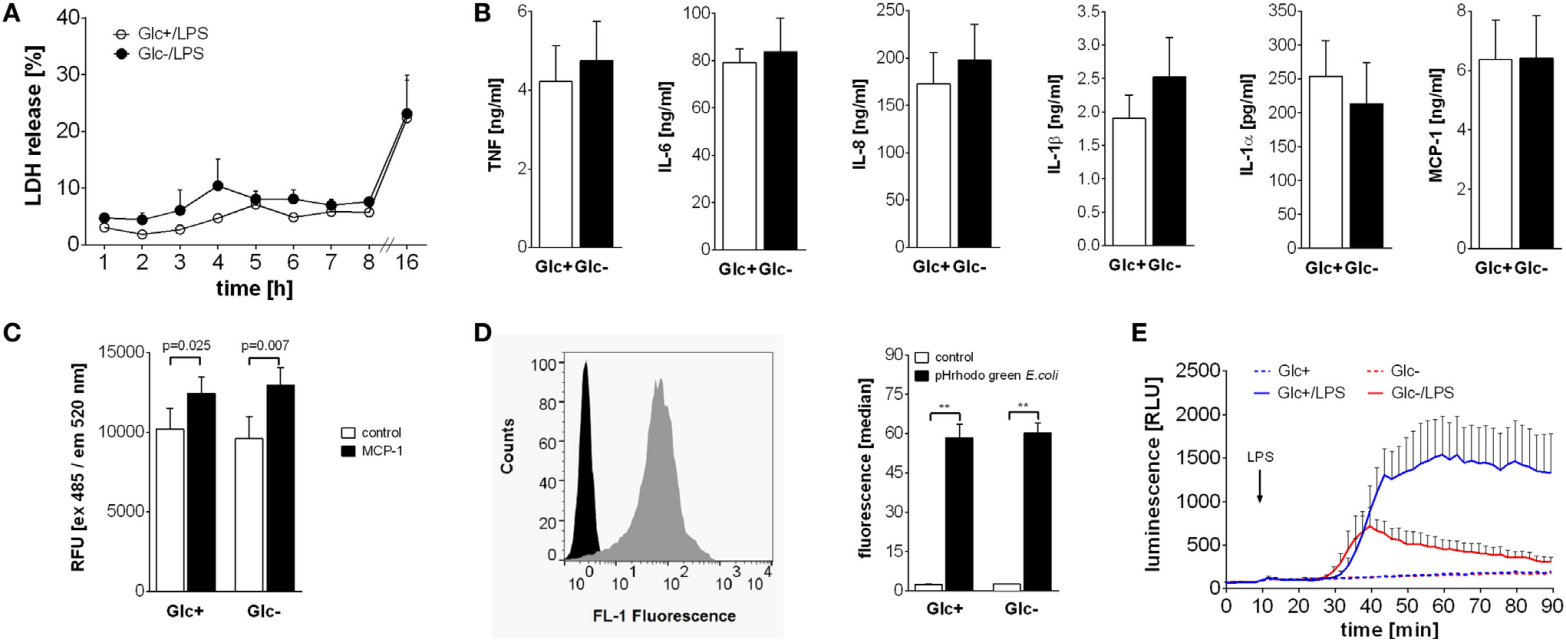
**Differential influence of glucose deprivation on monocyte functions**. **(A)** Cell death was determined by measuring the lactate dehydrogenase (LDH) release of monocytes cultured with or without glucose and is shown as percentage of total intracellular LDH. Depicted are mean values of *n* = 3 donors ± SEM. **(B)** Monocytes were stimulated with lipopolysaccharide (LPS) for 4 h (TNF) or 16 h (IL-6, IL-8, IL-1β, IL-1α, and MCP-1), and released cytokines were determined in the supernatant by ELISA. Shown are mean values (*n* = 4) ± SEM. **(C)** Migratory capacity of LPS-stimulated monocytes with or without glucose was determined in a Boyden chamber with MCP-1. Migrated cells were stained after 4 h with calcein AM, the fluorescence was determined and is shown as mean relative fluorescence units ± SEM (*n* = 6). **(D)** Phagocytic activity of 2 h lipopolysaccharide (LPS)-stimulated monocytes with or without glucose was analyzed with pHrodo Green *E. coli* BioParticles. Particles were added for 10 min, and the fluorescence of monocytes was determined by flowcytometry. The bar chart depicts the median fluorescence ± SEM of monocytes from *n* = 3 donors. **(E)** The oxidative burst after LPS stimulation of 2 h glucose-deprived and control monocytes was measured by luminescence increase of oxidized luminol. Shown is the mean luminescence (*n* = 3) ± SEM. Statistical analysis was performed using the paired Student’s *t*-test. Significant changes are indicated.

Activation of monocytes by LPS leads to a multitude of cellular consequences. The high energy demand is met by the switch to aerobic glycolysis in response to LPS. Therefore, we sought to determine if glucose-deprived monocytes are functionally impaired. First we determined the vitality of LPS-activated monocytes in the presence and absence of glucose in the culture media. As shown in Figure 4A, LPS-activated monocytes release similar concentrations of LDH, a marker of cell damage, into the culture supernatant under both conditions. To test cytokine and chemokine production, monocytes were cultured with LPS in the presence or absence of glucose. Glycolytic and glucose-deprived monocytes responded to LPS with a comparable production of the cytokines TNF, IL-6, IL-1β, IL-1α, and the chemokines IL-8 and MCP-1 (Figure 4B). Next we determined the chemotactic migration of monocytes to a gradient of MCP-1. As shown in Figure 4C, LPS-activated monocytes show similar rates of spontaneous and of MCP-1 directed migration in the presence and absence of glucose.

To test their phagocytic capability, monocytes were cultured for 2 h with LPS in the presence or absence of glucose and subsequently incubated with fluorescence-labeled *E. coli*. Phagocytosis was determined by flow cytometry. As shown in Figure 4D, phagocytosis rates of LPS-activated glycolytic and glucose-deprived monocytes are comparable. Finally, we tested the production and release of reactive oxygen species (ROS) from monocytes in response to LPS. The contribution of the NADPH oxidase to the OCR in LPS-activated monocytes was already shown in Figures 1H,I. Here, we measured the resulting respiratory burst with luminol-enhanced chemiluminescence. LPS induced a time-dependent increase in ROS production in glycolytic monocytes (Figure 4E). In contrast, glucose-deprived monocytes responded with a markedly decreased ROS production to LPS (Figure 4E).

### Active AMPK Drives Oxidative Phosphorylation while Inhibiting Respiratory Burst in Glucose-Deprived Monocytes

**Figure 5:**
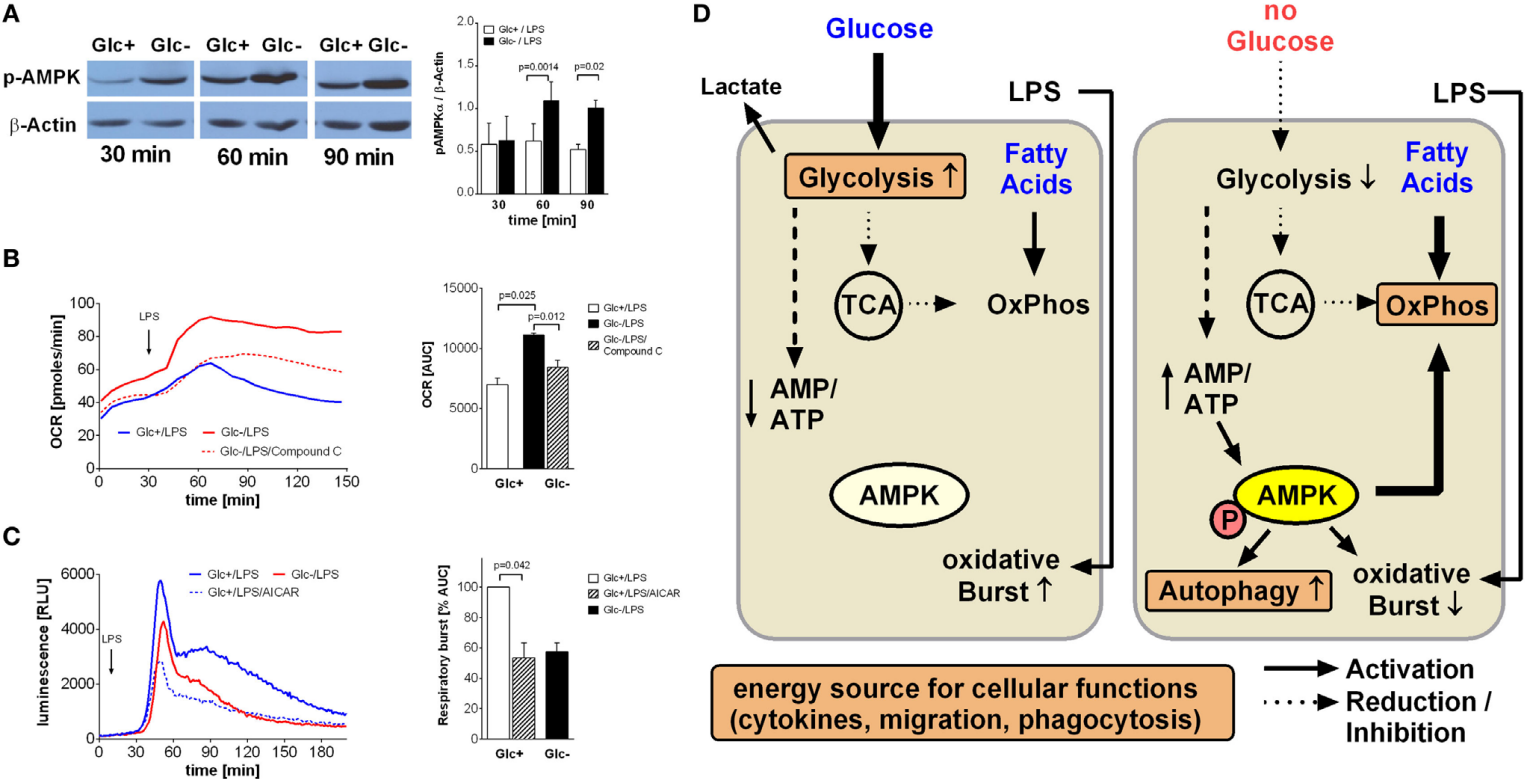
**AMP-activated protein kinase (AMPK) activation is responsible for increased oxidative phosphorylation and decreased respiratory burst of glucose-deprived monocytes**. **(A)** Activation of AMPK is demonstrated in Western Blot using anti-phosphoAMPKα Thr172 Antibody. Monocytes were stimulated for 30, 60, and 90 min with lipopolysaccharide (LPS) and with or without glucose. Shown is one representative Western Blot out of four independent experiments and the ratio of pAMPKα/β-actin densitometry quantification of *n* = 4. **(B)** The increase of oxidative phosphorylation due to glucose deprivation is mediated by AMPK activation. Monocytes under glucose deprivation were treated with the AMPK inhibitor compound C (10 µM; red dashed line) before the stimulation with LPS. Depicted are mean oxygen consumption rate curves and area under the curve (AUC) ± SEM from *n* = 3 donors. **(C)** The oxidative burst of LPS-stimulated monocytes is regulated by AMPK activation. Monocytes were cultured for 2 h with (blue) or without (red) glucose and then stimulated with LPS. Activation of AMPK in the presence of glucose with AICAR decreased the oxidation of luminol. Shown are mean luminescence rates and the calculated AUC ± SEM of *n* = 3 experiments. Statistical analysis was performed using the paired Student’s *t*-test. **(D)** Graphical summary of AMPK-mediated switch from glycolysis to oxidative phosphorylation under glucose-deprived conditions.

An increased AMP/ATP ratio as shown in Figure 2C is an activation signal for the cell energy sensor AMPK (5' AMPK). Accordingly, we determined AMPK activation in LPS-stimulated glycolytic and glucose-deprived monocytes by western blotting. As shown in Figure 5A, phosphorylation of AMPK on Thr172 is higher in LPS-activated glucose-deprived monocytes compared to glycolytic monocytes, indicating activation of the kinase.

In light of the aforementioned OCR increase following glucose deprivation, we sought to determine the contribution of AMPK activation to this increase. Compound C, a specific inhibitor of AMPK, led to a significant decrease in oxygen consumption in LPS-stimulated glucose-deprived monocytes, indicating inhibited oxidative phosphorylation (Figure 5B). This prompted us to study the influence of AMPK on the respiratory burst, which was the only monocyte function influenced by glucose deprivation. Indeed, we found that activation of AMPK by AICAR in LPS-stimulated glycolytic monocytes reduced their ROS production to levels similar to those observed in glucose-deprived monocytes (Figure 5C).

## Discussion

Monocytes and macrophages respond to LPS with a switch from oxidative phosphorylation to aerobic glycolysis, similar to the Warburg effect seen in cancer cells (5, 10, 29). We show here that in monocytes, glucose deprivation during activation with LPS leads to increased oxidative phosphorylation compensating at least partially for the increased energy demand during this activation phase.

Glycolysis only generates two molecules of ATP out of one molecule glucose whereas oxidative phosphorylation yields 36 ATP molecules (34). However, aerobic glycolysis is able to generate more ATP out of glucose than oxidative phosphorylation over short periods of time due to its faster conversion rate (35). In contrast to both modes of glucose metabolism, β-oxidation of one molecule long-chain fatty acid and further oxidation *via* TCA and oxidative phosphorylation results in an even higher ATP output (34). Our results show that the increased oxygen consumption in glucose-deprived monocytes is mainly required for fatty acid oxidation as demonstrated by the inhibition of oxygen consumption by the CPT-1 inhibitor Etomoxir. The attempt to compensate for glucose deprivation by fatty acid oxidation is only partially effective since we observed an increase in total AMP concentrations and in the AMP/ATP ratio in glucose-deprived monocytes during LPS stimulation.

The dependence on fatty acids of oxidative phosphorylation in LPS-stimulated, glucose-deprived monocytes is interesting because the cell culture medium RPMI 1640 does not contain added fatty acids or lipids. However, fetal calf serum contains free fatty acids and lipids. Lagarde et al. reported that medium with 20% fetal calf serum contains 74 nmol/ml free fatty acids and 197 nmol/ml total lipids (36). In our study, 10% fetal calf serum was added to the culture medium in most of the experiments, with the exception of the Seahorse Extracellular Flux analyses, which were performed with medium containing 5% fetal calf serum because 10% fetal calf serum disturbs the measurements. Altogether, only minimal lipid quantities were introduced to the monocyte culture in our experiments.

Another source of fatty acids for β-oxidation are LDs, which contain mostly triglycerides and cholesterol esters surrounded by a phospholipid layer (37–39). LPS is known to increase LD numbers in macrophages by enhancing fatty acid uptake and storage as triglycerides in LDs, while decreasing lipolysis and fatty acid oxidation (40). We could confirm a similar LD enrichment in LPS-stimulated monocytes. Following glucose deprivation, however, the frequency of LD+ monocytes and their phospholipid content was significantly lower. This might reflect the shift from fatty acid synthesis toward consumption of fatty acids *via* β-oxidation in glucose-deprived monocytes. To consume fatty acids, cells must mobilize them from LDs, which has been attributed to lipolysis, with LDs working as a conduit, delivering fatty acids directly into colocalized mitochondria (37). Importantly, AMPK, which we found to be activated in glucose-deprived monocytes, has been described to orchestrate the LD–mitochondria interactions to efficiently supply fatty acids for β-oxidation (38).

We also observed a minor contribution of glutamine utilization to oxygen consumption in glucose-deprived LPS-stimulated monocytes. It is known that resting murine macrophages show high glutamine utilization (31) and that LPS-stimulated murine bone marrow derived macrophages show an increase in intracellular succinate due to glutamine-dependent anaplerosis (4). Increased levels of succinate are known to stabilize the transcription factor HIF-1α, leading to transcription of IL-1β and glycolysis enzyme genes (4). The reported mediator of this stabilization is an increase in succinate levels. Since we found succinate levels unchanged following glucose deprivation, an additional contribution of HIF-1α stabilization in the forced switch to increased oxidative phosphorylation in monocytes appears unlikely.

AMP-activated protein kinase is the main cellular energy sensor (24). The energy status of the cell is sensed by monitoring the AMP/ATP ratio, and the resulting activation of AMPK leads to activation of catabolic and inhibition of anabolic pathways (24). Under glucose deprivation, we observed an increase in the AMP/ATP ratio and an activation of AMPK in LPS-stimulated monocytes. Accordingly, AMPK activation is likely to lead to increased fatty acid oxidation and decreased lipid synthesis under these conditions. Inhibition of AMPK activation was able to block the metabolic shift to oxidative phosphorylation, indicating that AMPK is indeed the pivotal regulator triggering the cellular switch (see graphical summary Figure 5D).

Despite glucose deprivation and decreased energy status of the cell, cytokine production, phagocytosis and chemotactic migration of monocytes remained intact. In addition, we observed no reduction in monocyte vitality under glucose deprivation. This is surprising because the switch to glycolysis in response to LPS is important for providing intermediates for nucleotide, amino acid and fatty acid synthesis *via* the pentose–phosphate pathway (6). The pentose–phosphate pathway is also important for the generation of reducing equivalents of NADPH (6). However, the forced metabolic switch to fatty acid oxidation seems to be able to compensate most of these processes. The observed activation of autophagy is likely a compensatory mechanism in response to glucose deprivation and activation of AMPK (33, 41). Autophagy removes damaged cellular components and provides nutrients by degradation of these components (33). In addition, autophagy is able to prevent an inadequate immune response by degrading inflammasome components (42).

Most of the monocyte effector functions were not affected by glucose deprivation. However, the LPS-induced NADPH oxidase-dependent respiratory burst was decreased in glucose-deprived monocytes. This inhibition was not due to limited availability of NADPH but was a direct effect of AMPK activation, since it could be reproduced by AICAR. It has been shown before that activation of AMPK leads to an inhibition of respiratory burst in neutrophils and other cells, probably by inhibition of p47phox phosphorylation and translocation to the plasma membrane (43–45). One might speculate that although most cellular functions remained intact, it is preferable for the starved monocyte to limit the generation of ROS and its undesirable effects on the cell itself.

In summary, we have demonstrated that circulating monocytes are metabolically equipped to perform pro-inflammatory immune functions under conditions of nutrient deprivation, in particular glucose deprivation, which are likely encountered under various pathological conditions. Their major metabolic strategy to compensate for the LPS-induced Warburg effect is the switch to fatty acid oxidation orchestrated by active AMPK.

## Ethics Statement

This study was carried out in accordance with the recommendations of “Ethik-Kommission an der Medizinischen Fakultät der Universität Leipzig” with written informed consent from all subjects.

## Author Contributions

NR performed most of the experiments and data analysis and was involved in drafting the manuscript. KF was responsible for performing the phagocytosis assay. SS performed data analysis on lipid droplets. SR and AK performed some of the Seahorse experiments. SB and MB performed measurement of intracellular polar metabolites using IC-MS/MS and Biocrates measurements. MK performed electron microscopy. UW and MR conceived of the project, were involved in data analysis, and drafted the manuscript.

## Conflict of Interest Statement

The authors declare that the research was conducted in the absence of any commercial or financial relationships that could be construed as a potential conflict of interest.

## Supplementary Material

Table S1 **Phosphatidylcholine concentrations determined with the Biocrates platform in lipopolysaccharide-stimulated monocytes at 1 and 6 h**.

Figure S1 **Apocynin inhibits the oxidative burst in primary monocytes**. **(A)** The contribution of NADPH oxidase to the oxygen consumption rate (OCR) of unstimulated monocytes was analyzed by injection of the specific inhibitor apocynin. Shown are mean OCR rates of *n* = 3 donors. **(B)** Inhibition of the lipopolysaccharide (LPS)-induced oxidative burst by apocynin in monocytes. Shown is the mean luminescence of one representative experiment.
