# Supplementary figures and images for: Fatty Acid Oxidation Compensates for Lipopolysaccharide-Induced Warburg Effect in Glucose-Deprived Monocytes
Source: Front Immunol. 2017 May 29;8:609. doi: 10.3389/fimmu.2017.00609 (PMC5447039; doi:10.3389/fimmu.2017.00609)

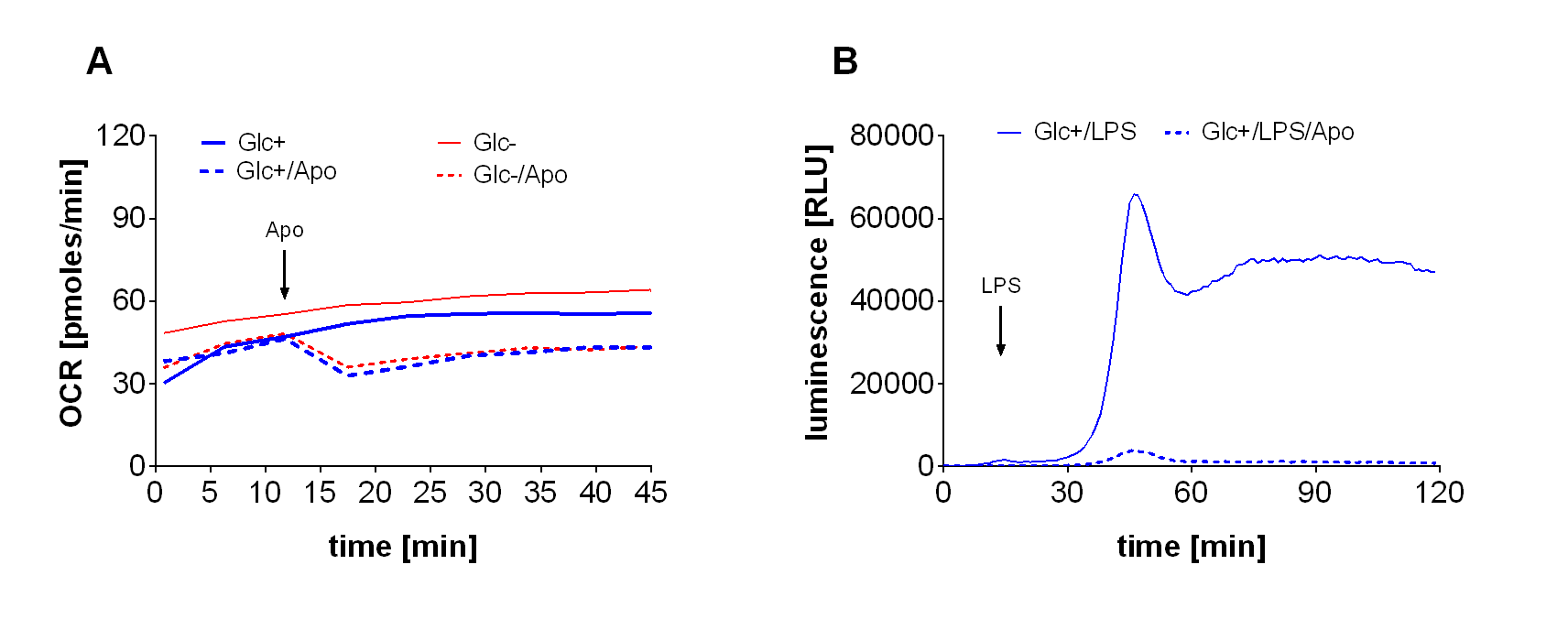

Supplement: Figure S1 — Apocynin inhibits the oxidative burst in primary monocytes. (A) The contribution of NADPH oxidase to the oxygen consumption rate (OCR) of unstimulated monocytes was analyzed by injection of the specific inhibitor apocynin. Shown are mean OCR rates of n = 3 donors. (B) Inhibition of the lipopolysaccharide (LPS)-induced oxidative burst by apocynin in monocytes. Shown is the mean luminescence of one representative experiment. [file Image_1.TIF]
